# Hb H disease resulting from the association of an α^0^-thalassemia allele [-(α)^20.5^] with an unstable α-globin variant [Hb Icaria]: First report on the occurrence in Brazil

**DOI:** 10.1590/S1415-47572009005000071

**Published:** 2009-12-01

**Authors:** Elza M. Kimura, Denise M. Oliveira, Kleber Fertrin, Valéria R. Pinheiro, Susan E. D. C. Jorge, Fernando F. Costa, Maria de Fátima Sonati

**Affiliations:** 1 Departamento de Patologia Clínica, Faculdade de Ciências Médicas, Universidade Estadual de Campinas, Campinas, SPBrazil; 2 Centro de Hematologia e Hemoterapia, Universidade Estadual de Campinas, Campinas, SPBrazil; 3 Centro Infantil Dr. Domingos A. Boldrini, Campinas, SPBrazil

**Keywords:** hereditary hemoglobinopathies, alpha-thalassemia, Hb H disease, Hb Icaria

## Abstract

Hb H Disease is caused by the loss or inactivation of three of the four functional α-globin genes. Patients present chronic hemolytic anemia and splenomegaly. In some cases, occasional blood transfusions are required. Deletions are the main cause of this type of thalassemia ( α-thalassemia). We describe here an unusual case of Hb H disease caused by the combination of a common α^0^ deletion [-( α) ^20.5^ ] with a rare point mutation (c.427T > A), thus resulting in an elongated and unstable α-globin variant, Hb Icaria, (X142K), with 31 additional amino-acid residues. Very high levels of Hb H and Hb Bart's were detected in the patient's red blood cells (14.7 and 19.0%, respectively). This is the first description of this infrequent association in the Brazilian population.

The alpha (α)-globin genes are duplicated (α_2_ and α_1_) and located on the short arm of chromosome 16 (16p13.3). Alpha-thalassemia (α-thal) is a hereditary condition resulting from deficient synthesis of α-globin chains. It has a worldwide distribution and reaches frequencies as high as 80% or more in some populations, reflecting positive selection after exposure to malaria (Higgs and Weatherall, 2009). Deletions resulting from unequal crossing-over between homologous sequences in the α-gene cluster are the main cause of this hemoglobinopathy and may affect one or both α genes in the haploid genome (α^+^ or α^0^ alleles, respectively). Nondeletional mutations are less frequent and usually correspond to more pronounced hematological alterations (Steinberg *et al.*, 2001; Weatherall and Clegg, 2001).

The combination of an α^0^ allele with an α^+^ allele, affecting three of the four functional α genes, leads to Hb H disease (—/-α), a moderate to severe chronic hemolytic anemia with the presence of 5%-25% of Hb H (β_4_) in peripheral blood erythrocytes (Steinberg *et al.*, 2001; Weatherall and Clegg, 2001). Hb H is unstable and precipitates in all circulating cells when submitted to any oxidant stress. Herein, we describe a rare case of Hb H disease resulting from the association of the [-(α)^20.5^] deletion, an α^0^ deletion commonly found in Mediterranean and Central Asian populations, with a point mutation (c.427T > A), this leading to the synthesis of an elongated and unstable α-globin variant, Hb Icaria [(X142K) modified C-terminal sequence: (142)Lys-Ala-Gly-Ala-Ser-Val-Ala-Val-Pro-Pro- Ala-Arg-Trp-Ala-Ser-Gln-Arg-Ala-Leu-Leu-Pro-Ser-Leu-His-Arg-Pro-Phe-Leu-Val-Phe-(172)Glu-COOH]. Hemoglobin Icaria, except for the residue at position 142, is similar to Hb Constant Spring and Hb Paksé (α_2_ 142, StopàGln and StopàTyr, respectively), which are frequent thalassemic alleles in southeastern Asia. The association of these variants with α^0^ alleles has been thoroughly studied as the cause of both Hb H disease and thalassemia intermedia (Schrier *et al.*, 1997; Wajcman *et al.*, 2008).

The patient here described is a 2-year-old boy of mixed Italian and African origin, followed up at Centro Infantil Dr. Domingos A. Boldrini, in Campinas, state of São Paulo, southeastern Brazil. He presented chronic hemolytic anemia, pallor, jaundice and spleen enlargement. Peripheral blood analysis revealed a remarkable degree of anisocytosis with microcytosis, hypochromia and 6.8% of reticulocytes. Serum ferritin was normal (45 ng/mL). The hematological data of the proband and his mother are summarized in Table 1. His father was not available for study.

Hb H, as well as its fetal version Hb Bart's - γ_4_, were both detected by alkaline electrophoresis and quantified by cation-exchange HPLC (High Performance Liquid Chromatography) (Variant II - β-Thalassemia Short Program; Bio-Rad Laboratories, Hercules, CA, USA). The percentages for Hb H and Hb Bart's were 14.7% and 19.0%, respectively (Figure 1). The presence of these abnormal variants was further confirmed by electrophoresis at neutral pH (Dacie *et al.*, 2006). Hemoglobin instability was demonstrated by n-butanol, isopropanol and heat tests. Heinz and Hb H inclusion bodies were observed in the patient's red blood cells (Dacie *et al.*, 2006) but no further anomalous hemoglobin was identified in his peripheral blood sample. No abnormal variant whatsoever was detected in the mother's blood sample.

Genomic DNA was obtained from peripheral blood leukocytes. Multiplex PCR for the most common α-thal alleles (Tan *et al.*, 2001) revealed the presence of the -(α)^20.5^ deletion in the patient's DNA sample (Figure 2), which was confirmed by specific gap-PCR (Kattamis *et al.*, 1996). The deletion removes a 20.5 kb fragment of DNA containing the entire α_2_ gene and part of the α_1_ gene, the latter, however, is not expressed (Steinberg *et al.*, 2001; Weatherall and Clegg, 2001).

Two α-globin genes still remained. Direct α-globin gene sequencing (ABI PRISM 377 DNA Automated Sequencer, Applied Biosystems, Foster City, CA, USA) with primers described elsewhere (Dodé *et al.*, 1990) identified base substitution (**T**AAà**A**AA) at the 142^nd^ (termination) codon of the α_2_-globin gene (Figure 3). This mutation, also found in the patient's mother, was confirmed by sequencing the opposite strand of the DNA, this resulting in an elongated (and unstable) α-chain constituted by 31 extra residues: (142)Lys-Ala-Gly-Ala-Ser-Val-Ala-Val-Pro- Pro-Ala-Arg-Trp-Ala-Ser-Gln-Arg-Ala-Leu-Leu-Pro-Ser-Leu-His-Arg-Pro-Phe-Leu-Val-Phe-(172)Glu-COOH. A stop codon was found at the new codon 173 (Hardison *et al.*, 2002).

**Figure 1:**
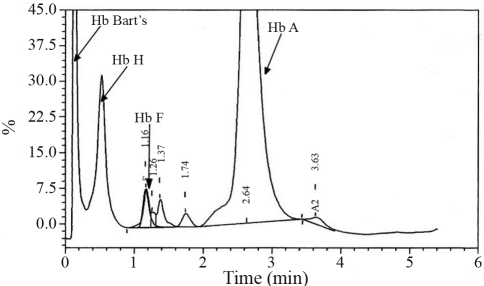
Cation-exchange HPLC chromatogram of the patient's blood sample showing hemoglobins Bart's and H beside the normal hemoglobins (Hb A_2_ and Hb A).

**Figure 2:**
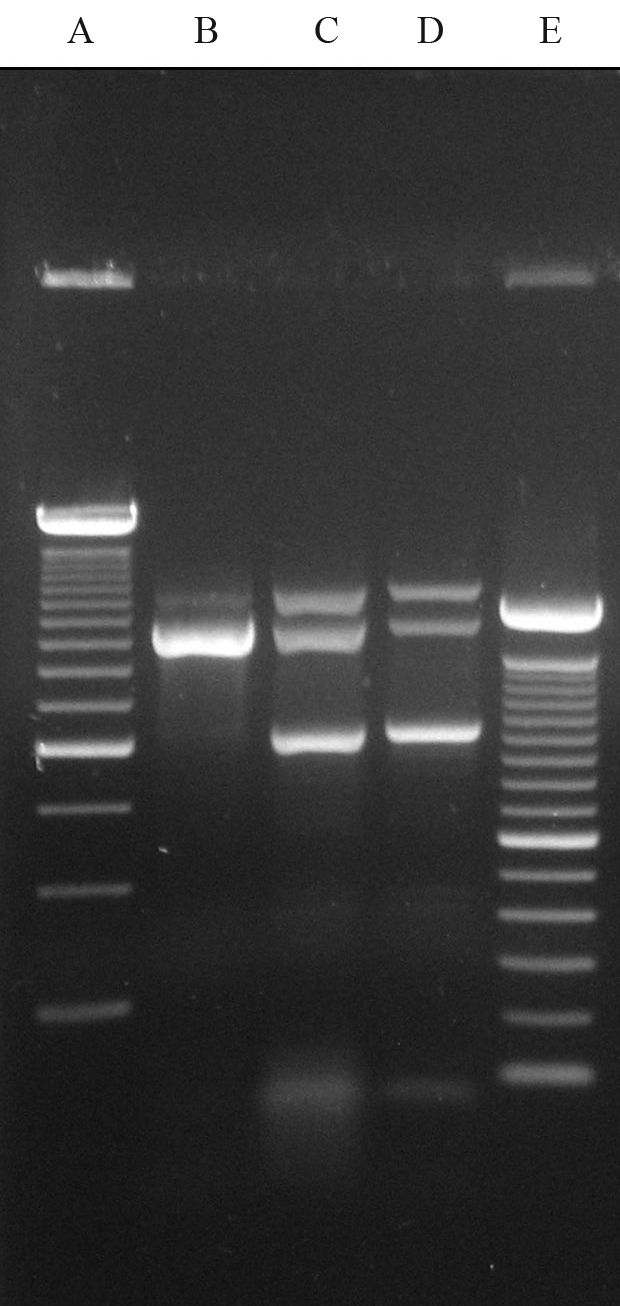
Multiplex PCR for screening of the most common α-thal alleles (Tan *et al.*, 2001).  A - 250 bp ladder marker; B - Normal Genotype Control (αα/αα); C - Patient [-(α)^20.5^/α^Hb Icaria^α]; D - Positive control for the -(α)^20.5^ deletion; E - 100 bp ladder marker.

**Figure 3:**
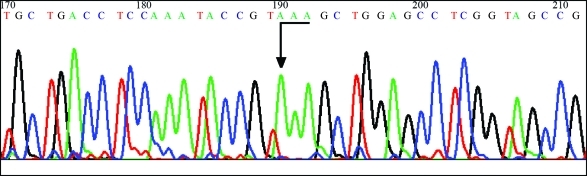
α_2_-globin gene sequencing identifying the Hb Icaria mutation (c.427T > A).

Hb Icaria is a rare Hb structural and thalassemic variant described in Greek, Yugoslavian and Macedonian families (Clegg *et al.*, 1974; Efremov *et al.*, 1990; Kanavakis *et al.*, 1996). It is difficult to detect in peripheral blood samples by the more commonly used techniques, due to its very low concentration and electrophoretic mobility, which is slower than that of Hb A_2_ at alkaline pH (Clegg *et al.*, 1974). The pathophysiology of these elongated chains has been attributed to mRNA instability (Waggoner and Liebhaber, 2003), but more recent studies have shown that it could be due to defective interaction with AHSP (alpha-hemoglobin stabilizing protein) (Turbpaiboon *et al.*, 2006). In the patient investigated here, the low availability of α-chains was probably responsible for the high levels of Hb H and Hb Bart's observed (33.7% of the total hemoglobin). Despite this, the alteration does not give rise to important clinical manifestations in heterozygous individuals, the case of our patient's mother, who has the αα/α^Hb Icaria^α genotype and is clinically silent.

This is the first description of Hb H disease caused by a combination of -(α)^20.5^ deletion with Hb Icaria [-(α)^20.5^/α^Hb Icaria^α] in the Brazilian population. It is also the first description of this variant in an individual of Italian and African origin. Our findings illustrate the importance of investigating these atypical cases and identifying their molecular basis and pathophysiological mechanisms. They also give us an idea of how frequent these mutations and associations are in our population.

## Figures and Tables

**Table 1:** Hematological data of the patient and his mother, both of African ancestry.

Hematological parameters	Patient	Mother
RBC (million/L)	4.57	4.74
Hb (g/dL)	7.6	12.9
Hct (%)	29.3	39.7
MCV (fL)	64.1	83.8
MCH (pg)	16.6	27.2
RDW-CV (%)	27.6	13.0
Reticulocytes (%)	6.8	2.0
Serum ferritin (ng/mL)	45.85	36.75
Hb Profile	A2 + A + Bart's + H	A2 + A
Hb Barts (%)	19.0	-
Hb H (%)	14.7	-
α-genotype	-(α)^20.5^/α^Hb Icaria^α	αα/α^Hb Icaria^α

RBC = Red Blood Cells; Hb = Hemoglobin; Hct = Hematocrit; MCV = Mean Corpuscular Volume; MCH = Mean Corpuscular Hemoglobin; RDW-CV = Coefficient of Variation of the Red Cell Distribution Width.
